# Incidence and predictors of left atrial appendage thrombus on transesophageal echocardiography before elective cardioversion

**DOI:** 10.1038/s41598-022-07428-5

**Published:** 2022-03-07

**Authors:** Felix K. Wegner, Robert Radke, Christian Ellermann, Julian Wolfes, Alicia J. Fischer, Helmut Baumgartner, Lars Eckardt, Gerhard-Paul Diller, Stefan Orwat

**Affiliations:** 1 Department of Cardiology II – Electrophysiology, University Hospital Muenster, Albert-Schweitzer-Campus 1, 48149 Muenster, Germany; 2 Department of Cardiology III – Adult Congenital and Valvular Heart Disease, University Hospital Muenster, Albert-Schweitzer-Campus 1, 48149 Muenster, Germany

**Keywords:** Cardiology, Risk factors, Neurological manifestations

## Abstract

Guidelines recommend transesophageal echocardiography (TEE) before cardioversion in thrombogenic arrhythmias when the requirement of ≥ 3 weeks of anticoagulation is not met. Current data to support this approach, especially with direct oral anticoagulants (DOAC), are scarce. We analyzed consecutive elective pre-cardioversion TEE in a high-volume electrophysiology center for the occurrence of left atrial appendage (LAA) thrombi or reduced LAA flow velocity. Possible predictors were recorded and compared in a multivariate logistic regression analysis. Consecutive pre-cardioversion TEE in 512 patients (148 female, median age 69 years) were included. In all patients, indication for TEE was either intake of anticoagulation < 3 weeks before cardioversion or uncertain adherence to the prescribed anticoagulation regimen. Of the 512 TEE, 19 (3.7%) depicted a LAA thrombus. An additional 41 patients (8.0%) showed either a reduced LAA flow velocity (≤ 20 cm/s), LAA sludge, or both. In a multivariate logistic regression analysis, QRS width on admission 12-lead ECG emerged as a possible predictor of LAA thrombus and reduced LAA flow (p = 0.008). Noteworthy, a high CHA_2_DS_2_-VASc score was not associated with an increased risk of reduced LAA emptying velocity and LAA thrombi were even found in patients with a CHA_2_DS_2_-VASc score of 0 (n = 1) and 1 (n = 1). The presence of LAA thrombus before an elective cardioversion is a rare event in the age of direct oral anticoagulants. However, LAA thrombi occurred even in supposed low-risk individuals according to the CHA_2_DS_2_-VASc score. QRS width may aid in identifying patients at risk of reduced LAA flow velocity.

## Introduction

For more than twenty years, guidelines on the management of atrial fibrillation (AF) have recommended transesophageal echocardiography (TEE) before an elective cardioversion (ECV) in patients who do not meet the requirement of uninterrupted effective oral anticoagulation for ≥ 3 weeks^1^. In the time since these recommendations were first given, the management of thromboembolic complications of atrial fibrillation has seen numerous changes including the establishment of the International Normalized Ratio^2^, the advent of the CHADS- and later the CHA_2_DS_2_-VASc scoring systems^3,4^, and the routine use of direct oral anticoagulants (DOAC)^5^. Nonetheless, stroke and peripheral embolic events remain a feared complication of atrial fibrillation, especially following cardioversion. The exclusion of a left atrial appendage (LAA) thrombus before cardioversion remains a cornerstone of guideline recommendations and clinical management by cardiologists and general internists alike^6^, although it is unclear whether the available data are still relevant in the changed landscape of modern atrial fibrillation management. The present study was thus designed to analyze the incidence of LAA thrombi in a contemporary cohort and elicit predictive factors of low-flow situations of the LAA, possibly identifying patient groups at such a low risk of LAA thrombus that the risks of TEE might outweigh the benefits.

## Methods

### Study design

**Figure 1:**
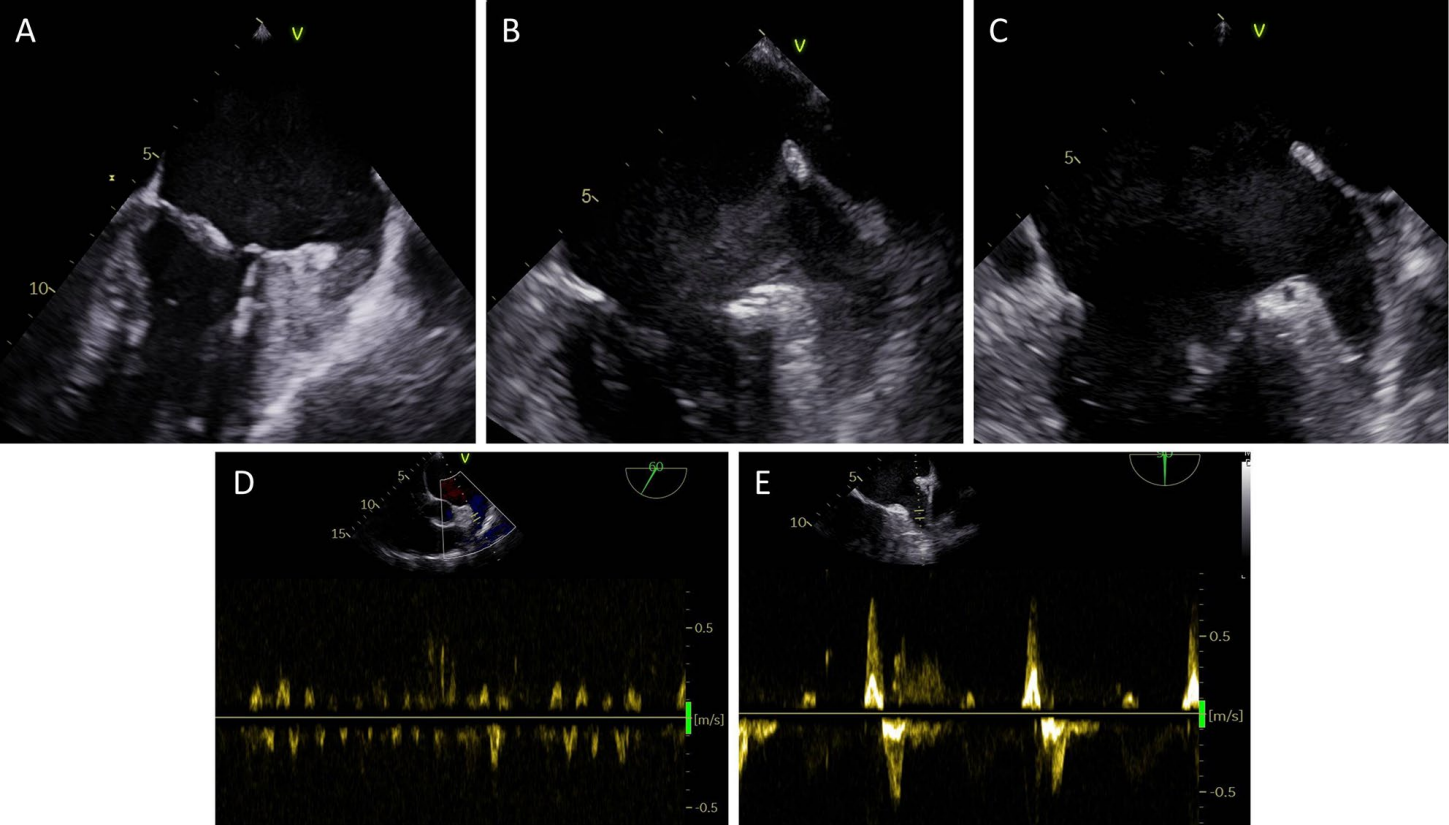
Representative images of a solid LAA thrombus (**A**), LAA sludge (**B**), not containing a solid thrombus on i.v. contrast imaging), and a LAA free of thrombus or sludge (**C**). (**D**) shows the PW Doppler signal in a patient with LAA emptying velocity reduced ≤ 20 cm/s while panel (**E**) shows a LAA with normal flow characteristics.

The present report was designed as a cohort study analyzing 512 consecutive TEE studies before a scheduled elective cardioversion between 2017 and 2020 in a high-volume electrophysiology center. All TEE performed before an ECV in this timeframe were included in the analysis. The study was conducted in accordance with the Declaration of Helsinki and all patients gave written informed consent for the planned TEE and cardioversion. As data analysis was retrospectively conducted on anonymized routine clinical data, no ethics committee approval was necessary as specified by the ethics committee of the Westfälische Wilhelms-Universität Münster. Clinical characteristics of included patients were recorded and compared in a multivariate logistic regression analysis (see statistical analysis) to elucidate possible predictors of low-flow situations in the LAA. In total, three patient groups were distinguished:

1. Patients with a solid LAA thrombus on TEE before scheduled ECV (Fig. 1A)
2. Patients with a low-flow state of the LAA, defined as:
  - LAA sludge without evidence of a solid thrombus (Fig. 1B) and/or
  - LAA emptying velocity ≤ 20 cm/s on PW-Doppler (Fig. 1D)
3. Patients without a LAA thrombus and with normal LAA flow characteristics (Fig. 1C,E).

### Transesophageal echocardiography

Transesophageal echocardiography was conducted according to established practice guidelines^7^. After excluding contraindications to TEE, all patients received intravenous sedation with propofol. Once adequate sedation was reached, the ultrasound probe was advanced to a mid-esophageal position and images of the LAA were obtained. The LAA was imaged in at least two planes (60° and 120°) with and without color doppler and blood flow velocity of the LAA was obtained using pulsed-wave doppler (PW). Additional imaging planes and the use of ultrasound contrast were at the discretion of the performing physician. Once sufficient images were obtained, the ultrasound probe was withdrawn and ECV was attempted if no thrombus was detected.

### Statistical analysis

Microsoft Excel© 2010 (Microsoft Corporation, Redmond, WA, USA) was used for data storage and SPSS Version 27 (IBM Corporation, Somers, NY, USA) for statistical analysis. Multivariate analysis was conducted using a multivariate logistic regression model including only factors with a significant association in a univariate analysis. Statistical significance was defined as a two-sided alpha level of 0.05 or less.

## Results

512 TEE studies were included in the analysis. Table 1 depicts the baseline characteristics of the patient population. All included patients underwent transesophageal echocardiography after giving informed consent. 292 (57%) of all patients were on a direct oral anticoagulant (DOAC) while 138 (27%) were anticoagulated with an oral vitamin K antagonist (VKA). The median INR of patients on VKA was 2.20 with an interquartile range (IQR) of 1.94–2.73. The median LVEF during pre-procedural TTE was 54% (IQR 40–60) and the median glomerular filtration rate (GFR, Cockcroft-Gault) was 78 ml/min (IQR 58–103 ml/min). The median EHRA score of all included patients was 2 (IQR 1–3). No severe complications occurred during any of the TEE studies and no patients had thromboembolic complications after ECV was conducted.

**Table 1:** Baseline characteristics of all patients.

Gender male/female	364/148 (71%/29%)
Age in years (IQR)	69 (59–78)
BMI in kg/m^2^ (IQR)	27.0 (24.2–31.2)
**Underlying rhythm**	
Atrial fibrillation	461 (90%)
Atrial flutter	51 (10%)
**CHA**_**2**_**DS**_**2**_**-VASc score**	
0–1	129 (25%)
2	114 (22%)
3	101 (20%)
4	82 (16%)
≥ 5	86 (17%)
**Cardiovascular comorbidities**	
Coronary heart disease	85 (17%)
Diabetes	84 (16%)
Hypertension	300 (59%)
Previous stroke	19 (4%)
Peripheral artery disease	30 (6%)
**Anticoagulation regimen**	
VKA	138 (27%)
Apixaban	130 (25%)
Rivaroxaban	74 (15%)
Edoxaban	67 (13%)
Dabigatran	21 (4%)
LMWH	2 (< 1%)
ASS	16 (3%)
None	64 (13%)

For age and BMI the median is given.

*IQR* interquartile range, *LMWH* low molecular weight heparin, *VKA* vitamin K antagonist.

### Incidence of LAA thrombus

19 TEE studies (3.7%) depicted a solid thrombus in the LAA. Clinical characteristics of this patient group are listed in Table 2. The median INR of the seven patients on VKA was 2.55 (IQR 1.95–3.07). Figure 1A shows a representative image of a LAA thrombus from one of the included patients. In all of these patients, the scheduled ECV was postponed. In eight patients, the anticoagulation regimen was changed as a result of the TEE study: Five patients were switched to dabigatran (three from apixaban, one from edoxaban, one not previously on oral anticoagulation), one patient was switched from dabigatran to VKA, one patient was switched from low molecular weight heparin to apixaban, and one further patient was switched from ASS to VKA. Of the remaining eleven patients, seven remained on VKA but were instructed on a close INR monitoring. Two patients each remained on apixaban and dabigatran and were instructed on medication adherence.

**Table 2:** Comparison between pre-cardioversion patients with normal LAA flow, patients with significantly reduced flow and patients with a LAA thrombus.

	Normal LAA flow (n = 452)	Reduced LAA flow (n = 41)	LAA thrombus (n = 19)	p-value (univariate analysis)	p-value (multivariate logistic regression)
Gender (m/f)	321 (71%) / 131 (29%)	30 (73%) / 11 (27%)	13 (68%) / 6 (32%)	0.92	
Age in years (IQR)	69 (58–77)	75 (63–82)	72 (60–77)	0.02	0.20
BMI in kg/m^2^ (IQR)	27 (24–31)	27 (24–31)	24 (23–33)	0.13	
**Underlying rhythm**					
Atrial fibrillation	406 (90%)	38 (93%)	18 (95%)	0.70	
Atrial flutter	46 (10%)	3 (7%)	1 (5%)		
CHA_2_DS_2_-VASc score	2.7 ± 1.7	3.0 ± 1.5	3.0 ± 1.9	0.80	
EHRA score	2.1 ± 0.8	2.0 ± 0.9	1.5 ± 0.7	0.39	
Coronary heart disease	75 (17%)	9 (22%)	1 (5%)	0.99	
Diabetes	74 (16%)	7 (17%)	3 (16%)	0.95	
Hypertension	267 (59%)	22 (54%)	11 (58%)	0.55	
Previous stroke	15 (3%)	2 (5%)	2 (11%)	0.21	
Peripheral artery disease	24 (5%)	4 (10%)	2 (11%)	0.15	
**Anticoagulation**					
DOAC	259 (57%)	24 (59%)	9 (47%)	0.47	
VKA	119 (26%)	12 (29%)	7 (37%)		
Heart rate in 1/min (IQR)	87 (70–105)	80 (63–100)	77 (59–93)	0.02	0.89
QRS width in ms (IQR)	100 (90–115)	110 (97–138)	115 (1–0–153)	0.004	**0.008**
LVEF in % (IQR)	55 (44–60)	45 (25–50)	35 (25–57)	0.01	0.63
GFR in ml/min (IQR)	79 (59–104)	64 (47–89)	80 (53–115)	0.10	

Statistical significance was calculated between the group with normal flow and a combination of the other two groups (n = 60) in a multivariate logistic regression analysis.

Significant values of the multivariate analysis are in bold.

### Incidence of reduced LAA flow

In 41 patients, the TEE study showed a low-flow situation of the LAA defined as either LAA sludge or a LAA emptying velocity ≤ 20 cm/s. Patient characteristics of this group are depicted in Table 2. The median INR of the twelve patients on VKA was 2.06 (IQR 1.91–2.74). Figure 1 shows representative images of both LAA sludge (panel B) and a reduced LAA emptying velocity (panel D). After exclusion of a solid thrombus, the scheduled ECV was conducted in all 41 patients. No thromboembolic complications occurred in any of the patients until hospital discharge.

### Predictors of LAA thrombus and reduced LAA flow

A multivariate logistic regression analysis was conducted to elicit possible predictors of LAA thrombi or a reduced LAA flow. For statistical power and to offset unclear medication adherence, we combined the groups with a solid LAA thrombus and with reduced LAA emptying velocity and compared these 60 patients with the remaining 452 patients with a normal LAA flow state. Univariate analyses showed a significant association between a reduced LAA flow velocity and age (p = 0.02), LVEF (p = 0.01), heart rate on admission (p = 0.02), and QRS (p = 0.004). In the multivariate logistic regression analysis, only QRS width remained significantly associated with a reduced LAA flow velocity or the presence of a LAA thrombus (p = 0.008, area under curve 0.78, see Table 2). The odds ratio per millisecond of QRS prolongation was 1.02 (95% CI 1.01–1.04). There was no association between reduced LAA flow velocity and a higher CHA_2_DS_2_-VASc score and LAA thrombi were found even in patients with a CHA_2_DS_2_-VASc score of 0 (n = 1) and 1 (n = 1).

## Discussion

The present study provides an update on the available evidence for LAA thrombus detection on transesophageal echocardiography before an elective cardioversion in a large cohort. We were able to show that the incidence of LAA thrombi before ECV was below 4% in this contemporary patient population with an additional 8% of patients displaying a significantly reduced LAA flow velocity. In a multivariate logistic regression model, QRS width was significantly associated with the presence of a LAA thrombus or reduced LAA flow velocity, which were combined for statistical analysis to increase power and to aid clinicians in detecting patients who should receive a thorough TEE examination before cardioversion.

In their seminal study evaluating the use of TEE published in 2001, Klein et al. reported an incidence of LAA thrombi of 13.8% in a patient population of 549 patients scheduled for elective cardioversion for AF^8^. In comparison, we were able to document a dramatically reduced incidence of 3.7%. Multiple factors may explain this difference. Firstly, atrial fibrillation management has undergone various changes in the time since Klein et al. published their results. Since the establishment of CHA_2_DS_2_-VASc score, more patients receive continuous effective oral anticoagulation than in previous decades. This is reflected by the fact that about 85% of our included patients were on effective anticoagulation, albeit either for < 3 weeks or with uncertain adherence. Additionally, DOAC are likely more effective anticoagulating agents than VKA and patients remaining on vitamin K antagonists may benefit from a closer monitoring, for example by utilizing home INR measurements. Furthermore, advances in ultrasound devices may enable echocardiographers to better visualize the LAA and by decreasing uncertainty allow more patients to be cardioverted. Our reported incidence also more closely resembles the very low incidence of LAA thrombi in patients before a pulmonary vein isolation procedure as reported recently in a study by Göldi et al.^9^. It should be noted, however, that these patients were younger (61 vs. 69 years) and comparatively healthier (e.g., CHA_2_DS_2_-VASc score of 1.8 vs. 2.7) than our included patient population.

Interestingly, our statistical analysis was unable to show an association between established risk factors for systemic embolism in atrial fibrillation such as the CHA_2_DS_2_-VASc score and reduced LAA velocities or LAA thrombus. In contrast, only QRS width remained significantly associated with a reduced LAA emptying velocity or thrombus in a multivariate logistic regression analysis. This may be an indicator that comorbidities more specific to the heart may play a larger role in the creation of LAA thrombi than in peripheral embolism. A slightly wider QRS during atrial fibrillation may indicate an impaired electrical conduction system or a subclinically diseased ventricular myocardium. Although they did not reach significance in the multivariate analysis, a low heart rate during atrial fibrillation and reduced LVEF were associated with a low LAA flow velocity or thrombus in a univariate analysis and would be aligned with this explanation. Importantly, a lack of power due to the comparatively low number of patients does not allow for the interpretation that reduced LVEF does not play a role in LAA thrombus formation. Further reports by other working groups are needed to confirm or refute these findings.

While the occurrence of a LAA thrombus before an elective cardioversion is a rare event, our data show that it presents in a very heterogeneous group of patients. Importantly, no single risk factor or established scoring system can identify a patient population without a relevant risk for a significantly reduced LAA flow velocity or thrombus. Since the risks associated with transesophageal echocardiography are minute^10^, our data support the use of TEE when the patient is not on anticoagulation or adherence is unclear.

### Limitations

As a single-center cohort study, the present report should only be viewed as hypothesis-generating and possible predictors of reduced LAA emptying velocities or LAA thrombi should be interpreted cautiously due to low patient numbers. Data on individual medication adherence before ECV was limited and thus no correlation could be drawn between the number of missed doses (in case of DOAC) or time outside of therapeutic INR (in case of VKA) and the occurrence of LAA thrombi. However, LAA emptying velocity is not influenced by anticoagulation regimen and thus the analysis for possible predictors of low LAA flow were unlikely to be affected.

## Conclusion

In a contemporary cohort of 512 patients, LAA thrombi were found in 3.7% of transesophageal echocardiography studies before a scheduled elective cardioversion. An additional 8% of patients had a significantly reduced LAA emptying velocity, representing a high-risk group for the formation of a LAA thrombus. In a multivariate logistic regression analysis, only QRS width was significantly associated with reduced LAA flow velocities. Interestingly, a high CHA_2_DS_2_-VASc score was not associated with reduced LAA flow. Our data support the routine use of TEE before ECV even in the changed landscape of modern atrial fibrillation treatment, as no patient groups were able to be identified in which the risks of TEE conceivably outweigh the benefits.
